# Supplementary material for: A fault-intrusion-tolerant system and deadline-aware algorithm for scheduling scientific workflow in the cloud
Source: PeerJ Comput Sci. 2021 Nov 2;7:e747. doi: 10.7717/peerj-cs.747 (PMC8576554; doi:10.7717/peerj-cs.747)
Supplement: Supplemental Information 6 [file peerj-cs-07-747-s006.zip › Myproject1.1/docs/org/cloudbus/cloudsim/distributions/UniformDistr.html]

UniformDistr


JavaScript is disabled on your browser.


- Overview
- Package
- Class
- Use
- Tree
- Deprecated
- Index
- Help

- Prev Class
- Next Class

- Frames
- No Frames

- All Classes

- Summary:
- Nested |
- Field |
- Constr |
- Method

- Detail:
- Field |
- Constr |
- Method


org.cloudbus.cloudsim.distributions

## Class UniformDistr

- java.lang.Object
- - org.cloudbus.cloudsim.distributions.UniformDistr

- All Implemented Interfaces:
  :   ContinuousDistribution

  ---

    

  ```
  public class UniformDistr
  extends java.lang.Object
  implements ContinuousDistribution
  ```

  A random number generator based on the Uniform distribution.

  Since:
  :   CloudSim Toolkit 1.0

- - ### Constructor Summary

    Constructors

    Constructor and Description

    `UniformDistr(double min,
    double max)`

    Creates new uniform distribution.

    `UniformDistr(double min,
    double max,
    long seed)`

    Creates new uniform distribution.
  - ### Method Summary

    Methods

    | Modifier and Type | Method and Description |
    | --- | --- |
    | `double` | `sample()` Generate a new random number. |
    | `static double` | `sample(java.util.Random rd, double min, double max)` Generates a new random number based on the number generator and values provided as parameters. |
    | `void` | `setSeed(long seed)` Set the random number generator's seed. |

    - ### Methods inherited from class java.lang.Object

      `clone, equals, finalize, getClass, hashCode, notify, notifyAll, toString, wait, wait, wait`

- - ### Constructor Detail


    - #### UniformDistr

      ```
      public UniformDistr(double min,
                  double max)
      ```

      Creates new uniform distribution.

      Parameters:
      :   `min` - minimum value
      :   `max` - maximum value


    - #### UniformDistr

      ```
      public UniformDistr(double min,
                  double max,
                  long seed)
      ```

      Creates new uniform distribution.

      Parameters:
      :   `min` - minimum value
      :   `max` - maximum value
      :   `seed` - simulation seed to be used
  - ### Method Detail


    - #### sample

      ```
      public double sample()
      ```

      Generate a new random number.

      **Specified by:**
      :   `sample` in interface `ContinuousDistribution`

      Returns:
      :   the next random number in the sequence


    - #### sample

      ```
      public static double sample(java.util.Random rd,
                  double min,
                  double max)
      ```

      Generates a new random number based on the number generator and values provided as
      parameters.

      Parameters:
      :   `rd` - the random number generator
      :   `min` - the minimum value
      :   `max` - the maximum value

      Returns:
      :   the next random number in the sequence


    - #### setSeed

      ```
      public void setSeed(long seed)
      ```

      Set the random number generator's seed.

      Parameters:
      :   `seed` - the new seed for the generator


- Overview
- Package
- Class
- Use
- Tree
- Deprecated
- Index
- Help

- Prev Class
- Next Class

- Frames
- No Frames

- All Classes

- Summary:
- Nested |
- Field |
- Constr |
- Method

- Detail:
- Field |
- Constr |
- Method
